# Racial and Ethnic and Rural Variations in Access to Primary Care for Veterans Following the MISSION Act

**DOI:** 10.1001/jamahealthforum.2024.1568

**Published:** 2024-06-21

**Authors:** Amy K. Rosen, Erin Beilstein-Wedel, Michael Shwartz, Heather Davila, Deborah Gurewich

**Affiliations:** 1 VA Boston Healthcare System, Center for Healthcare Organization, Implementation and Research, Boston, Massachusetts; 2 Department of Surgery, Boston University Chobian and Avedisian School of Medicine, Boston, Massachusetts; 3 VA Iowa City Health Care System, Iowa City; 4 Department of Internal Medicine, University of Iowa Carver College of Medicine, Iowa City; 5 Department of Internal Medicine, Boston University Chobian and Avedisian School of Medicine, Boston, Massachusetts

## Abstract

**Importance:**

The 2018 Veterans Affairs Maintaining Internal Systems and Strengthening Integrated Outside Networks (VA MISSION) Act was implemented to increase timely access to care by expanding veterans’ opportunities to receive Veterans Affairs (VA)–purchased care in the community (community care [CC]). Because health equity is a major VA priority, it is important to know whether Black and Hispanic veterans compared with White veterans experienced equitable access to primary care within the VA MISSION Act.

**Objective:**

To examine whether utilization of and wait times for primary care differed between Black and Hispanic veterans compared with White veterans in rural and urban areas after the implementation of the VA MISSION Act.

**Design, Setting, and Participants:**

This cross-sectional study used VA and CC outpatient and consult data from the VA’s Corporate Data Warehouse for fiscal years 2021 to 2022 (October 1, 2020, to September 30, 2022). Separate fixed-effects multivariable models were run to predict CC utilization and wait times. Each model was run twice, once comparing Black and White veterans and then comparing Hispanic and White veterans. Adjusted risk ratios (ARRs) were calculated for Black and Hispanic veterans compared with White veterans within rurality status for both outcomes.

**Main Outcomes and Measures:**

VA and CC primary care utilization as measured by primary care visits (utilization cohort); VA and CC primary care access as measured by mean wait times (access cohort).

**Results:**

A total of 5 046 087 veterans (994 517 [19.7%] Black, 390 870 [7.7%] Hispanic, and 3 660 700 [72.6%] White individuals) used primary care from fiscal years 2021 to 2022. Utilization increased for all 3 racial and ethnicity groups, more so in CC than VA primary care. ARRs were significantly less than 1 regardless of rurality status, indicating Black and Hispanic veterans compared with White veterans were less likely to utilize CC for primary care. There were 468 246 primary care consultations during the study period. The overall mean (SD) wait time was 33.3 (32.4) days. Despite decreases in wait times over time, primary care wait times remained longer in CC than in VA. Black veterans compared with White veterans had significantly longer wait times in CC (ARRs >1) but significantly shorter wait times in VA (ARRS <1) regardless of rurality status in VA and CC. CC wait times for Hispanic veterans compared with White veterans were longer in rural areas only and in VA rural and urban areas (ARRs >1).

**Conclusion and Relevance:**

The results of this cross-sectional study suggest that additional research should explore the determinants and implications of utilization differences among Black and Hispanic veterans compared with White veterans. Efforts to promote equitable primary care access for all veterans are needed so that policy changes can be more effective in ensuring timely access to care for all veterans.

## Key Points

**Question:**

How was increased access to community care (CC) within the Veterans Affairs Maintaining Internal Systems and Strengthening Integrated Outside Networks (VA MISSION) Act associated with primary care access among rural/urban Black and Hispanic veterans compared with White veterans?

**Findings:**

In this cross-sectional study of 5 046 087 veterans who used primary care, despite increases in veterans’ utilization of CC, Black and Hispanic veterans waited longer for primary care in CC compared with White veterans except in CC urban areas where Hispanic veterans had shorter wait times compared with White veterans (all significant differences).

**Meaning:**

The study results suggest that equitable access to primary care was not achieved with the VA MISSION Act and that a better understanding of the sources of health care disparities is needed so that strategies can be implemented to ensure that all veterans obtain timely care.

## Teaser

This cross-sectional study examines the utilization of and wait times for primary care for Black and Hispanic veterans compared with White veterans in rural and urban areas after the VA MISSION Act.

## Introduction

After long delays in outpatient care were reported in the Veterans Health Administration (VA) 2014 access crisis, Congress passed the Veterans Access Choice and Accountability Act (Choice Act; public law No. 113-146) to improve access to timely, high-quality care for veterans across all care settings.^1^ The Choice Act substantially increased options for eligible veterans to receive care outside the VA through use of community clinicians paid for by the VA. Passage of the 2018 Maintaining Internal Systems and Strengthening Integrated Outside Networks Act (MISSION Act; public law No. 115-182) further expanded the opportunity for veterans to receive VA-purchased care in the community (community care [CC]) through contracts with Community Care Network (CCN) providers managed by third-party administrators. In fiscal year (FY) 2022 alone, approximately 45% of 6.2 million veteran enrollees utilized CC for care.

Adoption of these policies precipitated a substantial transformation in the way the VA provides care, intensifying VA’s role as a purchaser of care. Although the Choice Act was intended as a short-term policy to address VA’s access crisis, the MISSION Act established a permanent CC program, requiring VA to build a network of CC clinicians to improve care access for veterans. Despite this potential benefit, concerns were raised about the unintended consequences of additional outsourcing (eg, fragmenting coordination of care and increasing attrition from VA care).^2,3,4,5,6,7^

Although veterans have historically relied on VA for primary care, utilization of CC for primary care steadily increased throughout the Choice Act period (FY 2015-FY 2018). Increases were associated with long delays in obtaining VA primary care, particularly among rural veterans who resided far from their local VA facility and/or who lived in areas with limited clinician supply.^6,8,9^ Sociodemographic characteristics (eg, Hispanic ethnicity, having additional health insurance, or lower disease burden) were also associated with utilization of CC for primary care.^8,9,10,11^ Despite increased utilization of CC for primary care, CC wait times were consistently longer than VA wait times during the Choice Act period, particularly for rural veterans.^8^

To our knowledge, few studies have examined the early association of MISSION Act implementation with primary and specialty care wait times (ie, time from when a medical consultation was requested to when the consultation appointment occurred). They generally corroborate Choice Act findings^6,12,13^ regarding longer wait times in CC compared with VA and also provide additional insights into geographic variation in wait times.^12^ However, it is unknown whether utilization of CC for primary care continued to increase during the later MISSION Act period (when more widespread adoption was likely to occur) and how that was associated with wait times. Since delayed access to primary care is associated with increased morbidity and mortality as well as decreased health care utilization and personal well-being, examining whether trends in primary care wait times have persisted in VA and CC is essential.^12,14,15,16^ Further, given reported wait time disparities for racial and ethnic minority groups for specialty care within the Choice and MISSION acts, it is important to determine if similar trends exist in primary care.^13,17^ Since health equity is a top VA priority, this information may help guide and support VA’s strong commitment to reducing disparities.^18,19,20,21^

To fill these gaps, we examined the extent to which utilization of and timely access to primary care were comparable for Black and Hispanic veterans in rural and urban areas compared with their White veteran counterparts. We expected that the MISSION Act would be associated with increased utilization of CC and improved access to VA and CC primary care but that Black and Hispanic veterans would experience longer CC wait times compared with White veterans, especially those living in rural settings.

## Methods

This study received a nonresearch determination from the VA Boston Healthcare Research Administration; thus, institutional review board approval and informed consent were waived. It followed the Strengthening the Reporting of Observational Studies in Epidemiology (STROBE) reporting guidelines for cross-sectional studies.

### Data Sources and Sample

This was a retrospective 2-year observational study using VA and CC outpatient and referral data obtained from VA’s Corporate Data Warehouse from FY 2021 to FY 2022 (October 1, 2020, to September 30, 2022). We used the Healthcare Common Procedure Coding System/*Current Procedural Terminology* (*CPT*) codes and clinician taxonomy codes to identify VA and CC primary care utilization; we also used place of service codes to identify CC primary care utilization (eTable 1 in Supplement 1 summarizes all codes used; eTables 2-5 in Supplement 1 provide specific coding details). Outpatient primary care consultations (ie, approved requests for primary care services in VA and/or CC) were identified using specific stop codes (identifiers that indicate the type of clinical encounter the veteran requested) (eTable 6 in Supplement 1).

In addition to obtaining the sociodemographic characteristics, medical conditions, and social risk factors of veterans from the Corporate Data Warehouse, we used the VA Planning Systems Support Group’s file to obtain veterans’ rurality.^22^ VA facility was identified from the 3- to 5-digit institution code associated with each primary care visit or consultation. Region was manually coded by aligning the state where each facility was located with its associated regional CCN (eFigure in Supplement 1).^23^

The study sample consisted of 2 distinct veteran cohorts: a utilization and an access cohort. The former was composed of 2 groups: (1) veterans who used VA exclusively for primary care (VA primary care users) and (2) veterans who had at least 1 primary care visit in CC (CC primary care users) during the study period. The latter cohort included all veterans who had an initial visit for primary care in either VA or CC during the study period. Follow-up primary care visits were excluded from the access cohort.

### Dependent Variables

The 2 outcome measures were primary care visit for the utilization cohort and consultation wait time (hereafter wait time) for the access cohort. Primary care visit (which could occur in person or through telemedicine) was coded dichotomously (VA/CC); multiple primary care visits on the same day were counted as 1 visit. Wait time was defined as the number of days from when a veteran’s clinician requested a primary care consultation to when the consultation appointment occurred.^24^ Because means and regression coefficients are sensitive to large outliers, we windsorized wait times; specifically, wait times in the top 1% of the distribution for both FYs combined (ie, consultations with wait times >179 days [n = 8410]) were set to 179 days. Both outcomes could occur either in VA and/or CC.

### Independent Variables

Two independent variables were of primary interest: (1) veterans’ race and ethnicity and (2) veterans’ rurality status. Additionally, veterans’ consultation setting of care was included as a dichotomous variable (VA/CC) in the access models. Race and ethnicity were based on self-identification and classified into 3 categories: Black non-Hispanic (Black), Hispanic, and White non-Hispanic (White).^25,26^ The rurality status of veterans was categorized into a dichotomous variable (rural/urban), combining rural with highly rural because of the few highly rural veterans in the sample (1.8% and 3.8% in the utilization and access cohorts, respectively).

Other independent variables associated with utilization and access included mean-centered age (coded continuously), sex (male/female/unknown), marital status (married/not married/unknown), social risk (housing instability and/or food insecurity, both coded as yes/no/unknown), the Gagne comorbidity score (based on the presence of 32 comorbidities),^27,28^ CCN region (categorized into 5 regions), FY (FY 2021 and FY 2022) and priority group (which we classified into 3 categories [1-3, 4-7, and 8]).^17,29,30,31,32^ Priority group indicates a veteran’s priority level for enrollment in VA and is based on specific eligibility criteria, including severity of service-connected disabilities and income.

### Analysis

We ran descriptive statistics on the characteristics of veterans in each cohort. Because a veteran could have more than 1 primary care visit and/or consultation during the study period, we reported unique veteran counts and means for the dependent and independent variables. We compared Black veterans with White veterans and Hispanic veterans with White veterans in rural and urban areas using standardized differences in means or proportions (ie, effect sizes [ES]). We interpreted ES as small (0.20), medium (0.50), and large (0.80).^33^ We also graphically depicted trends in unadjusted utilization for VA and CC primary care users and trends in unadjusted means by consultation setting and race and ethnicity categories during the study period, per quarter, and nationwide.

We then ran 2 fixed-effects multivariable regression models, adjusting for sociodemographic and clinical variables, rurality status, and race and ethnicity categories. Model 1 was a logistic model predicting CC primary care utilization. Model 2 was a linear model predicting wait times for primary care, which also included the consultation setting (VA/CC). We ran each set of models twice, first to compare Black veterans with White veterans and second to compare Hispanic veterans with White veterans. Standard errors were adjusted for clustering at the facility level. All regressions were conducted in R (version 4.1.2; R Foundation) using the statistics library.

Next, we followed the approach described by Kleinman and Norton^34^ to compare the ratio of adjusted outcomes (ie, adjusted risk ratios [ARRs]) for both cohorts (Black veterans compared with White veterans and Hispanic veterans compared with White veterans). The ARRs were calculated separately for rural and urban veterans. For example, to compare the risk of using CC for Black and White rural veterans, we calculated the mean of the predicted probabilities of using CC separately for each rural veteran, first assuming that the veteran was Black and second assuming that the veteran was White. The ARR for the rural analysis comparing Black veterans with White veterans was the ratio of the 2 means (ie, the risk ratio compared 2 groups with the same risk profile as in the sample of rural veterans, except for their race and ethnicity category). To determine if the ARRs represented statistically significant differences between groups (eg, Black rural veterans compared with White rural veterans), we used bootstrapping (with 1000 samples) to estimate the 95% CIs for the ARRs stratified by facility. If the 95% CIs did not include 1, we considered the ARRs to be statistically significant at the 95% level, indicating that differences between race and ethnicity categories were present.

## Results

### Characteristics of the Utilization and Access Cohorts

The utilization cohort included 5 046 087 unique veterans with primary care visits in VA or CC during the study period; 19.7% were Black, 7.8% were Hispanic, and 72.6% were White. Most veterans (92.5%) received primary care exclusively in VA; only 7.5% of veterans had at least 1 CC primary care visit (Table 1). Most sociodemographic comparisons between veteran groups had ES around 0.2 or less, indicating small differences (eTable 7 in Supplement 1). Notable differences, in which ES was greater than 0.3, included age (in which Black and Hispanic veterans were younger than their respective White veteran counterparts in rural and urban areas), and CCN region (eg, Hispanic veterans living in rural and urban areas were less likely to reside in CCN region 2 [the Midwest] compared with urban White veterans).

**Table 1.** Key Measures of the Utilization and Access Cohorts^a^

Characteristic	Veterans, No. (%)														
	Overall	Urban^b^							Rural^b^						
		Black	Hispanic	White	Black compared with White veterans		Hispanic compared with White veterans		Black	Hispanic	White	Black compared with White veterans		Hispanic compared with White veterans	
					Effect size	*P* value	Effect size	*P* value				Effect size	*P* value	Effect size	*P* value
**Utilization cohor**t^c^^,^^d^															
Distinct veterans	5 046 087	830 574 (16.5)	331 388 (6.6)	2 160 539 (41.82)	NA	NA	NA	NA	163 943 (3.25)	59 482 (1.18)	1 500 161 (29.73)	NA	NA	NA	NA
Care setting															
VA primary care users	4 666 227 (92.5)	789 731 (95.1)	311 502 (94.0)	2 024 544 (93.7)	0.060	<.001	0.012	<.001	150 221 (91.6)	53 513 (89.9)	1 336 716 (89.1)	0.086	<.001	0.028	<.001
CC primary care users	379 860 (7.5)	40 843 (4.9)	19 886 (6.0)	135 995 (6.3)	0.060	<.001	0.012	<.001	13 722 (8.4)	5969 (10.0)	163 445 (10.9)	0.086	<.001	0.028	<.001
No. of VA visits per veteran, mean (SD)	3.05 (3.44)	3.44 (3.98)	3.22 (3.50)	3.06 (3.48)	0.140	<.001	0.045	<.001	3.11 (3.24)	2.75 (2.91)	2.79 (3.03)	0.102	<.001	0.013	<.001
No. of CC visits per veteran, mean (SD)	0.20 (1.15)	0.12 (0.89)	0.14 (0.93)	0.16 (1.08)	0.039	<.001	0.015	<.001	0.22 (1.13)	0.26 (1.27)	0.31 (1.37)	0.075	<.001	0.038	.002
Access cohort^e^^,^^f^															
Distinct veterans	386 764	67 977 (17.6)	28 845 (7.5)	153 826 (39.8)	NA	NA	NA	NA	11 828 (3.1)	4946 (1.3)	119 342 (30.9)	NA	NA	NA	NA
No. of consultations	468 246	82 730	33 607	185 264	NA	NA	NA	NA	13 985	5965	146 695	NA	NA	NA	NA
Wait time, mean (SD), d	33.31 (32.39)	31.07 (29.94)	39.01 (34.05)	31.57 (30.76)	0.016	<.001	0.229	<.001	35.28 (33.76)	39.89 (36.12)	34.98 (34.7)	0.009	.37	0.138	<.001
Consultation setting															
VA	324 636 (83.9)	63 887 (93.9)	26 929 (93.4)	140 807 (91.5)	0.095	<.001	0.069	<.001	9373 (79.2)	3358 (67.9)	80 282 (67.3)	0.273	<.001	0.013	.37
CC	62 128 (16.1)	4090 (6.0)	1916 (6.6)	13 019 (8.5)	0.095	<.001	0.069	<.001	2455 (20.8)	1588 (32.1)	39 060 (32.8)	0.273	<.001	0.013	.37

Abbreviations: CC, community care; NA, not applicable; VA, Veterans Health Administration.

^a^ Five VA facilities were excluded that had transitioned to Cerner, the new VA electronic health record system, due to lack of validated study measures at these sites.

^b^ Rurality was derived from the VA Planning Systems Support Group, which utilizes the definition of rurality from the Office of Management and Budget (urban, rural, and highly rural) based on Rural-Urban Commuting Area Codes.

^c^ Veterans with unknown rurality status (9.3%) and those who resided on an island (0.04%) were excluded from the utilization cohort.

^d^ A total of 328 501 patients (5%) were excluded from the utilization cohort because they were classified as “unknown” (ie, those cases that could not be classified due to missing race/ethnicity data, or reported as declined or unknown).

^e^ Veterans with unknown rurality status (3.9%) and those who resided on an island (0.8%) were excluded from the access cohort.

^f^ A total of 31 880 patients (7%) were excluded from the access cohort because they were classified as having unknown race and ethnicity (ie, those individuals who could not be classified due to missing race and ethnicity data, or self-reported as declined or unknown).

There were a total of 468 246 primary care consultations (among 386 764 veterans) in the access cohort. About 16% of all consultations were referred to CC. Rural Black veterans were less likely to have their primary care consultations in CC compared with rural Hispanic and White veterans (ES, 0.27). The overall unadjusted mean (SD) wait time across all consultations was 33.3 (32.4) days; unadjusted mean weight times were about 6 days higher than this for urban and rural Hispanic veterans (Table 1). Characteristics of the access cohort were similar to those of the utilization cohort (eTable 8 in Supplement 1).

### Trends in Primary Care Utilization and Access

**Figure 1.**
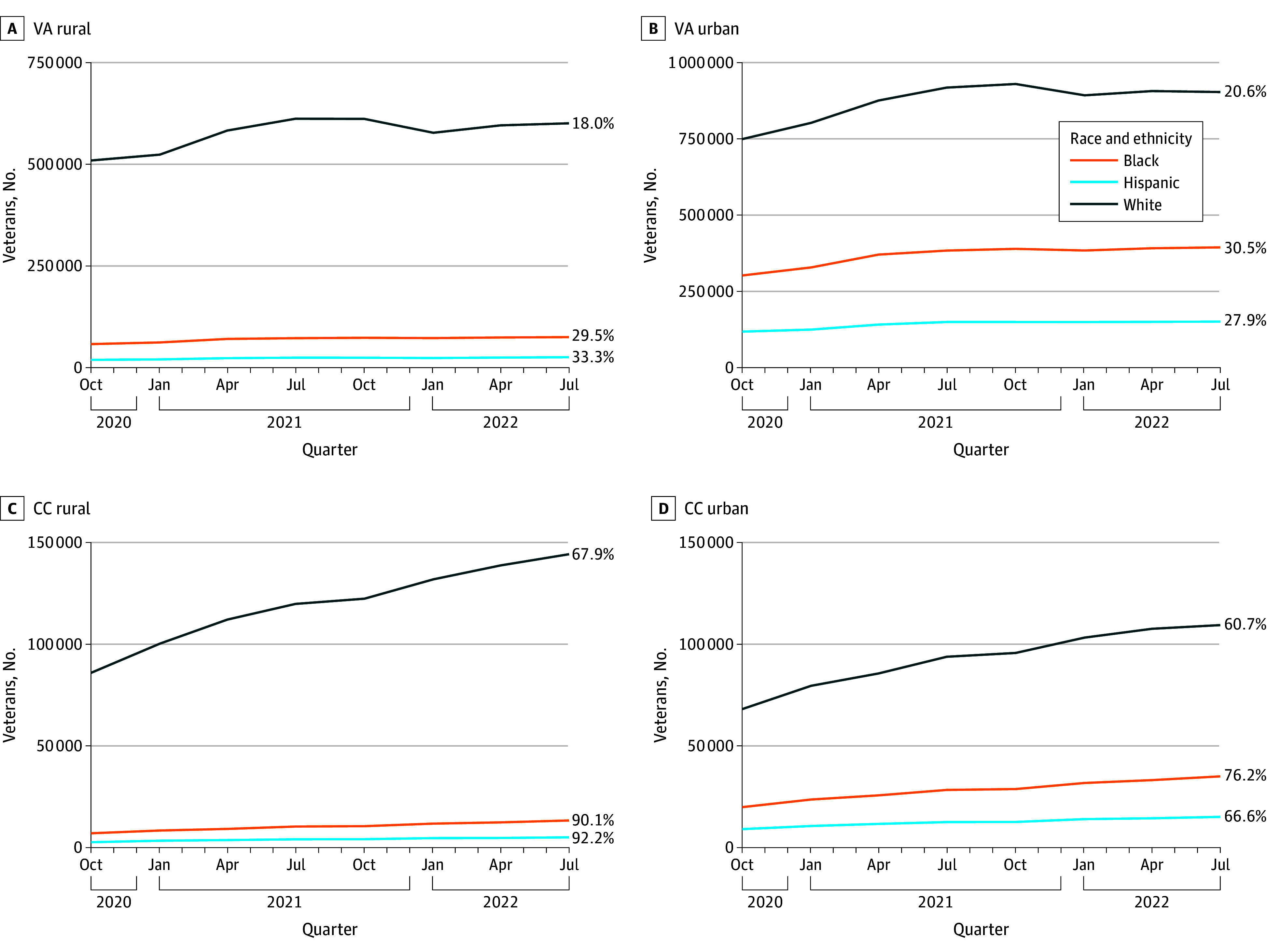
Health Care Utilization of Veterans per Quarter by Setting, Rurality Status, and Race and Ethnicity CC indicates community care; VA, US Department of Veterans Affairs.

Utilization of primary care increased during the study period among all groups regardless of rurality status or setting (VA/CC) (Figure 1; eFigure in Supplement 1). The percentage increases from the study’s start to end dates (October 1, 2020, to September 2022) in the number of veterans utilizing CC (ranging from 60.7%-92.2%) were larger than the percentage increases in those utilizing VA (18.0%-33.3%). However, because many more veterans used VA for primary care (4 666 227) compared with CC (379 860), small percentage increases in VA translate into much larger increases in the number of veterans using VA than large percentage increases in CC.

**Figure 2.**
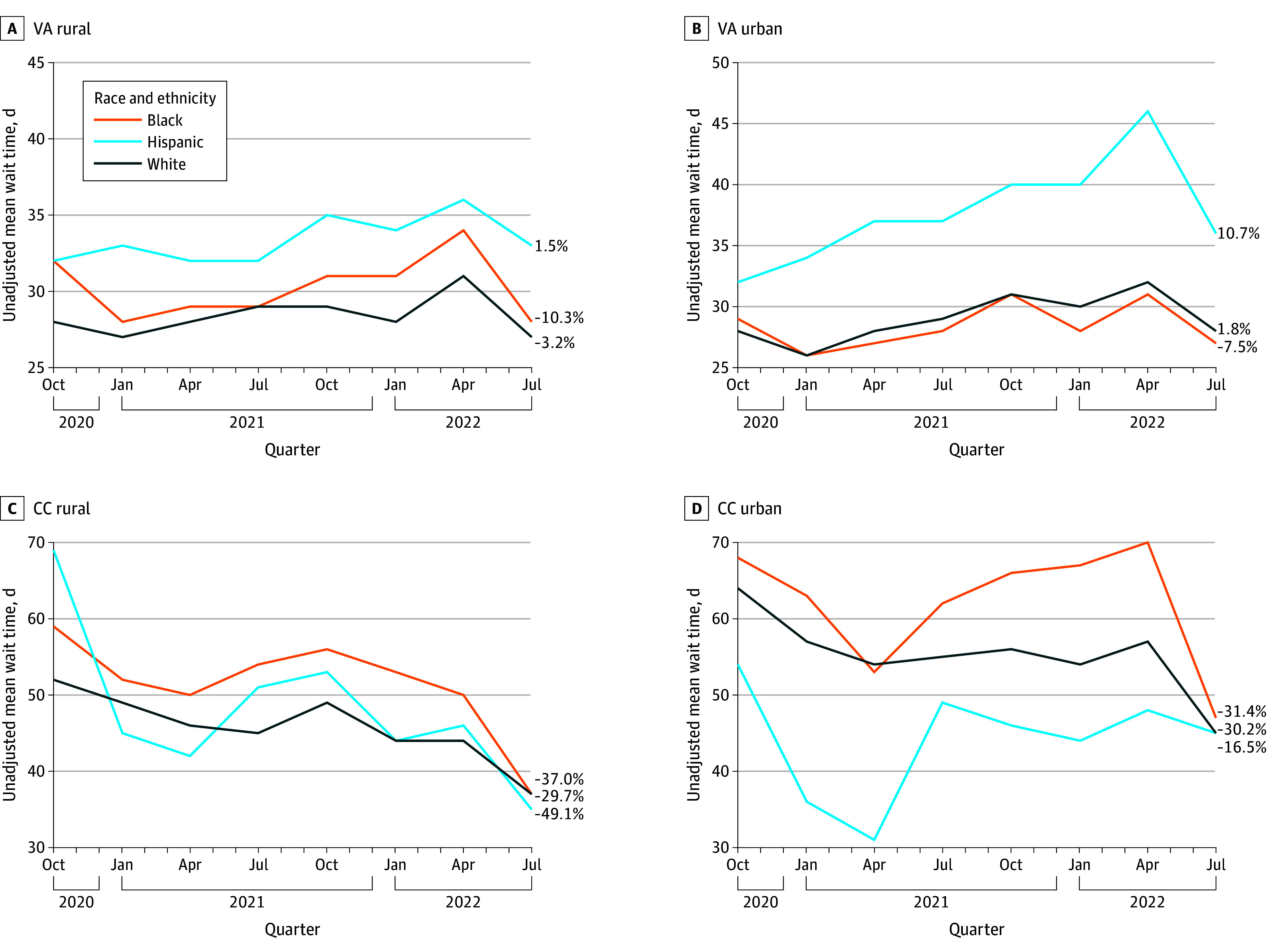
Unadjusted Mean Wait Times by Setting, Rurality Status, and Race and Ethnicity CC indicates community care; VA, US Department of Veterans Affairs.

Despite large percentage increases in utilization, there were substantial percentage decreases from baseline in mean unadjusted wait times for veterans using CC for all race and ethnicity categories (−16.4% to −49.1%) (Figure 2). In VA, results varied by race and ethnicity category and rurality. For example, there were percentage decreases for Black veterans in rural (−10.1%) and urban (−7.6%) areas, although there were small percentage increases for Hispanic veterans in urban (10.5%) and rural (1.5%) areas. Despite the greater percentage changes in wait times in CC, for all race and ethnicity categories, regardless of rurality status, CC mean wait times for primary care consultations remained consistently longer than those in VA. Five of the race and ethnicity categories experienced wait times of 35 days or shorter in VA, whereas all of the race and ethnicity categories had wait times of 35 days or longer in CC.

### Predicting Any CC Primary Care Utilization With ARRs

Black (Black veterans compared with White veterans) and Hispanic (Hispanic veterans compared with White veterans) veteran ARRs were statistically significant and less than 1 regardless of rurality status, indicating that CC utilization among Black veterans and White veterans was lower compared with White veterans (Table 2). For example, the ARR for Hispanic veterans compared with White veterans living in urban areas was 0.78 (95% CI, 0.77-0.79), indicating that urban Hispanic veterans compared with urban White veterans would, on average, be 78% as likely to use CC.

**Table 2.** Prediction of Any Community Care Utilization With ARRs^a^

Rurality^b^^,^^c^	ARR (95% CI)	
	Black veterans and White veterans^d^	Hispanic veterans and White veterans^e^
Rural	0.705 (0.695-0.714)	0.754 (0.738-0.769)
Urban	0.686 (0.680-0.692)	0.777 (0.767-0.787)

Abbreviation: ARR, adjusted risk ratio.

^a^ A total of 328 501 patients (5%) were excluded from the utilization cohort because they were classified as having unknown race and ethnicity (ie, those individuals who could not be classified due to missing race and ethnicity data, or self-reported as declined or unknown).

^b^ Rurality was derived from the VA Planning Systems Support Group, which utilizes the definition of rurality from the Office of Management and Budget (urban, rural, and highly rural) based on rural-urban commuting area codes.

^c^ Veterans with unknown rurality status (9.3%) and those residing on an island (0.04%) were excluded.

^d^ For the comparison between Black and White veterans, the ARR is Black veterans/White veterans.

^e^ For the comparison between Hispanic and White veterans, the ARR is Hispanic veterans/White veterans.

### Prediction of VA and CC Wait Times With ARRs

Wait time ARRs for Black veterans and Hispanic veterans were also statistically significant (Table 3). Rural and urban Black veterans compared with White veterans had longer primary care wait times in CC. For example, the ARR for urban Black veterans compared with White veterans in CC was 1.11 (95% CI, 1.08-1.14), indicating that urban Black veterans would have waited about 10% longer in CC than urban White veterans. Conversely, rural and urban Black veterans had shorter wait times in VA compared with White veterans. ARRs were less than 1 (eg, the ARR for urban Black veterans compared with White veterans in VA was 0.95; 95% CI, 0.94-0.95), indicating that urban Black veterans compared with urban White veterans would have waited, on average, about 5% less time in VA.

**Table 3.** Prediction of Mean Wait Times With ARRs^a^

Consultation setting	Rurality^b^^,^^c^	ARR (95% CI)	
		Black veterans and White veterans^d^	Hispanic veterans and White veterans^e^
CC	Urban	1.106 (1.078-1.135)	0.774 (0.743-0.805)
CC	Rural	1.052 (1.020-1.085)	1.046 (1.005-1.086)
VA	Urban	0.946 (0.938-0.954)	1.215 (1.201-1.229)
VA	Rural	0.971 (0.951-0.991)	1.128 (1.095-1.160)

Abbreviations: ARR, adjusted risk ratio; CC, community care; VA, Veterans Health Administration.

^a^ A total of 31 880 patients (7%) were excluded from the utilization cohort because they were classified as having unknown race (ie, those individuals who could not be classified due to missing race and ethnicity data, or self-reported as declined or unknown).

^b^ Rurality was derived from the VA Planning Systems Support Group, which utilizes the definition of rurality from the Office of Management and Budget (urban, rural, and highly rural) based on Rural-Urban Commuting Area codes.

^c^ Veterans with unknown rurality status (3.9%) and those residing on an island (0.08%) were excluded.

^d^ For comparison of Black and White veterans, the ARR is Black veterans/White veterans.

^e^ For the comparison between Hispanic and White veterans, the ARR is Hispanic veterans/White veterans.

Rural Hispanic veterans using CC also had longer wait times compared with rural White veterans (ARR, 1.05); in contrast, urban Hispanic veterans using CC had shorter wait times (ARR, 0.77). ARRs were greater than 1 for rural and urban Hispanic veterans using the VA (1.13 and 1.22, respectively), indicating longer average wait times compared with rural and urban White veterans.

## Discussion

To our knowledge, this is the first study to examine whether utilization of and timely access to primary care were comparable across race and ethnicity categories within urban and rural areas in the post–MISSION Act period. This study revealed several findings. First, as expected, we found substantial percentage increases in the number of veterans utilizing CC primary care during this period, which was consistent with prior studies.^9,11^ Despite the comparatively modest percentage increases in veterans’ utilization of VA, the much larger number of veterans using VA vs CC for primary care during the MISSION Act period indicated that reliance on VA for primary care continued to remain strong, mitigating concerns that the MISSION Act would contribute to privatization of VA care.^5,6,9^

Second, we found some interesting differences in utilization among race and ethnicity categories. Although Black and Hispanic veterans had higher percentage increases in CC utilization compared with White veterans, both groups were less likely to use CC for primary care than White veterans, regardless of rurality status. Further research is needed to understand whether the lower likelihood of using primary care in CC among Black and Hispanic Veterans was attributable to patient-level factors (eg, patient preferences), facility-level factors (eg, appointment scheduling practices), or system-level factors (eg, policies and legislation). If these results indicate factors that place Black and Hispanic veterans at a disadvantage, VA leadership will need to address this, given its strong commitment to achieving equitable access to care.^18,19,20,21^

Third, as expected, CC unadjusted mean wait times decreased substantially during the MISSION Act period for all veterans; however, CC unadjusted mean wait times remained consistently longer than those in VA, which was similar to findings from other studies and other outpatient care services.^6,12,17^ VA and CC unadjusted mean wait times exceeded the 20-day wait time standards established in the MISSION Act.^35^ Because primary care plays a key role in providing preventive care and managing and coordinating veterans’ health care,^9,10,36^ reducing wait times is essential for optimizing veterans’ overall health and well-being. VA policies and innovative strategies, such as the Referral Coordination Initiative (recently launched to increase veterans’ awareness of their options for when and where to receive care [VA or CC]),^37^ are needed to reduce the sources of delay for primary care in both settings.

Fourth, despite progress in reducing wait times, Black and Hispanic veterans experienced longer wait times in CC for primary care compared with White veterans (which was consistent with earlier results on disparities in specialty care).^13,17^ However, urban Hispanic veterans in CC had shorter wait times than White veterans. Aside from this one exception, these results indicated that inequitable access to primary care may have been a potential unintended consequence of the MISSION Act. Understanding the sources of health care disparities (eg, the unavailability of CC clinicians in areas where many Black and Hispanic veterans live) could help provide guidance on how to reduce these disparities and remove access barriers. Substantial investment in primary care that is focused on increasing accessibility (one of primary care’s core elements) may also lead to improvements in access and health care equity.^38,39^

### Limitations

This study had several limitations. Although we followed VA’s recommended methods for objectively measuring VA wait times,^24^ we lacked a subjective measure of access (ie, veterans’ perceptions of primary care access). The association between how long a veteran waits for an appointment and their perception of access and how this varies by racial and ethnic groups is not well understood. Finally, primary care is one of the least well-defined services; it can be defined multiple ways (eg, using stop/procedure codes, clinician taxonomies) that can vary between studies and health care systems.^6,9,12,24,40^

## Conclusions

Providing all veterans timely, equitable care access is a key VA priority.^18^ This study provides additional evidence that although the MISSION Act was associated with reduced wait times for primary care, it did not meet VA’s access standards. As increasing numbers of veterans utilize CC, any unintended consequences (eg, access disparities) associated with this policy shift need to be understood and addressed. Additional strategies are also needed to ensure that veterans obtain timely, equitable access to care in whatever setting they choose. Our findings may apply to other health care organizations that contract out care and are faced with similar challenges.

## Notes

Supplement 1. eTable 1. Comparison of Approach to Measuring Primary Care Utilization across Community and VA Settings using Administrative Data for Utilization Cohort eTable 2. List and Definition of Primary Care Current Procedural Codes (CPT) and Healthcare Common Procedure Coding System (HCPCS) Codes eTable 3. List and Definition of Primary Care Provider Taxonomy Codes eTable 4. Place of Service Claims Used to Identify Primary Care Utilization in Community Care Outpatient Claims eTable 5. List and Definition of VA Stop Codes Excluded from Primary Care Utilization Measure eTable 6. List of Stopcodes Included for Measuring Primary Care Access eFigure. VA Community Care Regional Networks eTable 7. Utilization Cohort Demographics eTable 8. Access Cohort Demographics

Supplement 2. Data sharing statement
