# Supplementary material for: Racial and Ethnic and Rural Variations in Access to Primary Care for Veterans Following the MISSION Act
Source: JAMA Health Forum. 2024 Jun 21;5(6):e241568. doi: 10.1001/jamahealthforum.2024.1568 (PMC11193128; doi:10.1001/jamahealthforum.2024.1568)
Supplement: Supplement 1. — eTable 1. Comparison of Approach to Measuring Primary Care Utilization across Community and VA Settings using Administrative Data for Utilization Cohort eTable 2. List and Definition of Primary Care Current Procedural Codes (CPT) and Healthcare Common Procedure Coding System (HCPCS) Codes eTable 3. List and Definition of Primary Care Provider Taxonomy Codes eTable 4. Place of Service Claims Used to Identify Primary Care Utilization in Community Care Outpatient Claims eTable 5. List and Definition of VA Stop Codes Excluded from Primary Care Utilization Measure eTable 6. List of Stopcodes Included for Measuring Primary Care Access eFigure. VA Community Care Regional Networks eTable 7. Utilization Cohort Demographics eTable 8. Access Cohort Demographics [file jamahealthforum-e241568-s001.pdf]

## Supplemental Online Content

Rosen AK, Beilstein-Wedel E, Shwartz M, Davila H, Gurewich D. Racial and ethnic and rural variations in access to primary care for veterans following the MISSION Act. *JAMA Health Forum*. 2024;5(6):e241568. doi:10.1001/jamahealthforum.2024.1568

**eTable 1.** Comparison of Approach to Measuring Primary Care Utilization across Community and VA Settings using Administrative Data for Utilization Cohort
**eTable 2.** List and Definition of Primary Care *Current Procedural Codes* (*CPT*) and Healthcare Common Procedure Coding System (HCPCS) Codes
**eTable 3.** List and Definition of Primary Care Provider Taxonomy Codes
**eTable 4.** Place of Service Claims Used to Identify Primary Care Utilization in Community Care Outpatient Claims
**eTable 5.** List and Definition of VA Stop Codes Excluded from Primary Care Utilization Measure
**eTable 6.** List of Stopcodes Included for Measuring Primary Care Access
**eFigure.** VA Community Care Regional Networks
**eTable 7.** Utilization Cohort Demographics
**eTable 8.** Access Cohort Demographics

This supplemental material has been provided by the authors to give readers additional information about their work.

© 2024 Rosen AK et al. *JAMA Health Forum*.

**eTable 1. Comparison of Approach to Measuring Primary Care Utilization across Community and VA Settings using Administrative Data for Utilization Cohort**

| Type of Code | VA | CC |
|---|---|---|
| CPT and HCPCS | 0500F, 99201, 99202, 99203, 99204, 99205, 99385, 99386, G0101, G0245, G0246, G0248, G0344, G0402, 0502F, 0503F, 1000F, 1001F, 2000F, 95115, 95117, 99058, 99211, 99212, 99213, 99214, 99215, 99354, 99355, 99366, 99367, 99395, 99396, 99401, 99402, 99403, 99404, 99411, 99412, 99420, 96060, 96161, 99429, G0247, G0250, G0420, G0421, G0463, 99024, 99241, 99242, 99243, 99244, 99245, 99271, 99272, 99273, 99274, 99275, 2001F, 2002F, 2004F, 2010F, 2014F, 2015F, 2016F, 2018F, 2040F, 2050F, 2027F, 2030F, 2031F, 99387, 99397 | (Same as VA) |
| Taxonomy | 363L00000X, 163WA2000X, 164W00000X, 163W00000X, 163WP2201X, 163WW0101X, 163WC0400X, 163WC1500X, 163WG0000X, 163WH0200X, 163WW0000X, 363A00000X, 363AM0700X, 364SC1501X, 364SF0001X, 364SH0200X, 364SW0102X, 363LA2200X, 363LF0000X, 363LP2300X, 363LS0200X, 363LW0102X, 203BF0100Y, 203BG0301Y, 203BG0000Y, 203BG0302Y, 203BI0300Y, 203BA0002Y, 203BG0303Y, 203BP0500Y, 390200000X, 203BA0001N, 203BG0300N, 207Q00000X, 207QA0000X 207QA0505X, 207QG0300X, 208D00000X, 207R00000X, 207RA0000X, 207RG0300X, 2083P0901X, 163WG0600X, 364SG0600X, 363LG0600X, 208000000X, 163WP0200X, 364SP0200X, 363LP0200X, 2083X0100X, 208M00000X, 2080A0000X, 2083P0500X | (Same as VA) |
| Place Of Service | N/A | 2, 3, 4, 5, 6, 7, 8, 9, 10, 11, 12, 15, 17, 18, 19, 20, 22, 24, 26, 27, 28, 29, 30, 35, 36, 37, 38, 39, 40, 43, 44, 45, 46, 47, 48, 49, 50, 52, 53, 55, 56, 57, 59, 62, 63, 64, 71, 72, 99 |
| Stop Codes (EXCLUDED) | 108, 119, 121, 190, 191, 213, 222, 443, 444, 445, 446, 447, 448, 450, 555, 568, 574, 651, 652, 653, 656, 658, 669, 681, 682, 130, 297 | N/A |
| VA = Veterans Health Administration<br>CC = Community Care | | |

© 2024 Rosen AK et al. *JAMA Health Forum.*

**eTable 2. List and Definition of Primary Care Current Procedural Codes (CPT) and Healthcare Common Procedure Coding System (HCPCS) Codes**

| CPT/HCPCS | Definition |
|---|---|
| 0500F | Patient Management |
| 99201 | New Patient Office or Other Outpatient Services |
| 99202 | New Patient Office or Other Outpatient Services |
| 99203 | New Patient Office or Other Outpatient Services |
| 99204 | New Patient Office or Other Outpatient Services |
| 99205 | New Patient Office or Other Outpatient Services |
| 99385 | New Patient Preventive Medicine Services |
| 99386 | New Patient Preventive Medicine Services |
| G0101 | Well Woman Exam |
| G0245 | Physician Evaluation and Management |
| G0246 | Physician Evaluation and Management |
| G0248 | HCPCS |
| G0344 | Initial Preventive Physical Examination |
| G0402 | Initial Preventive Physical Examination |
| 0502F | Patient Management |
| 0503F | Patient Management |
| 1000F | Patient History |
| 1001F | Patient History |
| 2000F | Physical Examination |
| 95115 | Professional Services |
| 95117 | Professional Services |
| 99058 | Miscellaneous Medicine Services |
| 99211 | Office or Other Outpatient Services |
| 99212 | Office or Other Outpatient Services |
| 99213 | Office or Other Outpatient Services |
| 99214 | Office or Other Outpatient Services |
| 99215 | Office or Other Outpatient Services |
| 99354 | Prolonged Services |
| 99355 | Prolonged Services |
| 99366 | Case Management Services |
| 99367 | Case Management Services |
| 99395 | Established Patient Preventive Medicine Services |
| 99396 | Established Patient Preventive Medicine Services |
| 99401 | Preventive Medicine Services |
| 99402 | Preventive Medicine Services |
| 99403 | Preventive Medicine Services |
| 99404 | Preventive Medicine Services |

© 2024 Rosen AK et al. *JAMA Health Forum.*

**eTable 2 (continued). List and Definition of Primary Care Current Procedural Codes (CPT) and Healthcare Common Procedure Coding System (HCPCS) Codes**

| CPT/HCPCS | Definition |
|---|---|
| 99411 | Preventive Medicine Services |
| 99412 | Preventive Medicine Services |
| 99420 | Health and Behavior Assessment |
| 96060 | Health and Behavior Assessment |
| 96161 | Health and Behavior Assessment |
| 99429 | Other Preventive Medicine Services |
| G0247 | HCPCS |
| G0250 | HCPCS |
| G0420 | HCPCS |
| G0421 | HCPCS |
| G0463 | HCPCS |
| 99024 | Miscellaneous Medicine Services |
| 99241 | New or Established Patient Office or Other Outpatient Consultation Services |
| 99242 | New or Established Patient Office or Other Outpatient Consultation Services |
| 99243 | New or Established Patient Office or Other Outpatient Consultation Services |
| 99244 | New or Established Patient Office or Other Outpatient Consultation Services |
| 99245 | New or Established Patient Office or Other Outpatient Consultation Services |
| 99271 | Confirmatory Consultations |
| 99272 | Confirmatory Consultations |
| 99273 | Confirmatory Consultations |
| 99274 | Confirmatory Consultations |
| 99275 | Confirmatory Consultations |
| 2001F | Physical Examination |
| 2002F | Physical Examination |
| 2004F | Physical Examination |
| 2010F | Physical Examination |
| 2014F | Physical Examination |
| 2015F | Physical Examination |
| 2016F | Physical Examination |
| 2018F | Physical Examination |
| 2040F | Physical Examination |
| 2050F | Physical Examination |
| 2027F | Physical Examination |
| 2030F | Physical Examination |
| 2031F | Physical Examination |
| 99387 | Preventive Medicine Services |
| 99397 | Preventive Medicine Services |

© 2024 Rosen AK et al. *JAMA Health Forum*.

**eTable 3. List and Definition of Primary Care Provider Taxonomy Codes**

| X12 Provider Taxonomy Code | Description |
|---|---|
| 163WA2000X | NURSING SERVICE; REGISTERED NURSE;ADMINISTRATOR |
| 164W00000X | NURSING SERVICE; LICENSED PRACTICAL NURSE |
| 163W00000X | NURSING SERVICE; REGISTERED NURSE |
| 163WP2201X | NURSING SERVICE; REGISTERED NURSE; AMBULATORY CARE |
| 163WW0101X | NURSING SERVICE; REGISTERED NURSE; WOMEN HEALTH CARE, AMBULATORY |
| 163WC0400X | NURSING SERVICE; REGISTERED NURSE; CASE MANAGEMENT |
| 163WC1500X | NURSING SERVICE; REGISTERED NURSE; COMMUNITY HEALTH |
| 163WG0000X | NURSING SERVICE; REGISTERED NURSE; GENERAL PRACTICE |
| 163WH0200X | NURSING SERVICE; REGISTERED NURSE; HOME HEALTH |
| 163WW0000X | NURSING SERVICE; REGISTERED NURSE; WOUND CARE |
| 363A00000X | PHYSICIAN ASSISTANTS&ADVANC. PRACTICE NURSING; PHYSICIAN ASSISTANT |
| 363AM0700X | PHYSICIAN ASSISTANTS&ADVANC. PRACTICE NURSING; PHYSICIAN ASSISTANT; MEDICAL |
| 364SC1501X | PHYSICIAN ASSISTANTS & ADVANCED PRACTICE NURS; CLINICAL NURSE SPECIALIST; COMMUNITY HEALTH/PUBLIC HEALTH |
| 364SF0001X | PHYSICIAN ASSISTANTS & ADVANCED PRACTICE NURS; CLINICAL NURSE SPECIALIST; FAMILY HEALTH |
| 364SH0200X | PHYSICIAN ASSISTANTS & ADVANCED PRACTICE NURS; CLINICAL NURSE SPECIALIST; HOME HEALTH |
| 364SW0102X | PHYSICIAN ASSISTANTS & ADVANCED PRACTICE NURS; CLINICAL NURSE SPECIALIST; WOMENS HEALTH |
| 363L00000X | PHYSICIAN ASSISTANTS & ADVANCED PRACTICE NURS; NURSE PRACTITIONER |
| 363LA2200X | PHYSICIAN ASSISTANTS & ADVANCED PRACTICE NURS; NURSE PRACTITIONER; ADULT HEALTH |
| 363LF0000X | PHYSICIAN ASSISTANTS & ADVANCED PRACTICE NURS; NURSE PRACTITIONER; FAMILY |
| 363LP2300X | PHYSICIAN ASSISTANTS & ADVANCED PRACTICE NURS; NURSE PRACTITIONER; PRIMARY CARE |
| 363LS0200X | PHYSICIAN ASSISTANTS & ADVANCED PRACTICE NURS; NURSE PRACTITIONER; SCHOOL |
| 363LW0102X | PHYSICIAN ASSISTANTS & ADVANCED PRACTICE NURS; NURSE PRACTITIONER; WOMENS HEALTH |
| 203BF0100Y | PHYSICIANS (M.D. AND D.O.); PHYSICIAN/OSTEOPATH; FAMILY PRACTICE |
| 203BG0301Y | PHYSICIANS (M.D. AND D.O.); PHYSICIAN/OSTEOPATH; GERIATRIC MEDICINE:FAMILY PRACTIC |
| 203BG0000Y | PHYSICIANS (M.D. AND D.O.); PHYSICIAN/OSTEOPATH; GENERAL PARCTICE |
| 203BG0302Y | PHYSICIANS (M.D. AND D.O.); PHYSICIAN/OSTEOPATH; GERIA. MEDICINE:GENERAL PRACTICE |
| 203BI0300Y | PHYSICIANS (M.D. AND D.O.); PHYSICIAN/OSTEOPATH; INTERNAL MEDICINE |

© 2024 Rosen AK et al. *JAMA Health Forum.*

**eTable 3 (continued). List and Definition of Primary Care Provider Taxonomy Codes**

| X12 Provider Taxonomy Code | Description |
|---|---|
| 203BA0002Y | PHYSICIANS (M.D. AND D.O.); PHYSICIAN/OSTEOPATH; ADOLESCENT MEDICINE:INTER.MEDICIN |
| 203BG0303Y | PHYSICIANS (M.D. AND D.O.); PHYSICIAN/OSTEOPATH; GERIATRIC MEDICINE:INTER.MEDICINE |
| 203BP0500Y | PHYSICIANS (M.D. AND D.O.); PHYSICIAN/OSTEOPATH; PREVENTIVE MEDICINE, GENERAL |
| 390200000X | PHYSICIANS (M.D.) AND OSTEOPATHS (D.O.); RESIDENT, OSTEOPATHIC |
| 203BA0001N | PHYSICIANS (M.D. AND D.O.); PHYSICIAN/OSTEOPATH; ADOLESCENT MEDICINE:FAMILY PARCTI |
| 203BG0300N | PHYSICIANS (M.D. AND D.O.); PHYSICIAN/OSTEOPATH; GERIATRIC MEDICINE |
| 207Q00000X | ALLOPATHIC AND OSTEOPATHIC PHYSICIANS; FAMILY PRACTICE |
| 207QA0000X | ALLOPATHIC AND OSTEOPATHIC PHYSICIANS; FAMILY PRACTICE; ADOLESCENT MEDICINE |
| 207QA0505X | ALLOPATHIC AND OSTEOPATHIC PHYSICIANS; FAMILY PRACTICE; ADULT MEDICINE |
| 207QG0300X | ALLOPATHIC AND OSTEOPATHIC PHYSICIANS; FAMILY PRACTICE; GERIATRIC MEDICINE |
| 208D00000X | ALLOPATHIC AND OSTEOPATHIC PHYSICIANS; GENERAL PRACTICE; |
| 207R00000X | ALLOPATHIC AND OSTEOPATHIC PHYSICIANS; INTERNAL MEDICINE; |
| 207RA0000X | ALLOPATHIC AND OSTEOPATHIC PHYSICIANS; INTERNAL MEDICINE; ADOLESCENT MEDICINE |
| 207RG0300X | ALLOPATHIC AND OSTEOPATHIC PHYSICIANS; INTERNAL MEDICINE; GERIATRIC MEDICINE |
| 2083P0901X | ALLOPATHIC AND OSTEOPATHIC PHYSICIANS; PREVENTIVE MEDICINE; PUBLIC HEALTH AND GENERAL PREVENTIVE MEDICINE |
| 163WG0600X | Nursing Service Providers; Registered Nurse; Gerontology |
| 364SG0600X | Physician Assistants & Advanced Practice Nursing Providers; Clinical Nurse Specialist; Gerontology |
| 363LG0600X | Physician Assistants & Advanced Practice Nursing Providers; Nurse Practitioner; Gerontology |
| 208000000X | Allopathic & Osteopathic Physicians; Pediatrics |
| 163WP0200X | Nursing Service Providers; Registered Nurse; Pediatrics |
| 364SP0200X | Physician Assistants & Advanced Practice Nursing Providers; Clinical Nurse Specialist; Pediatrics |
| 363LP0200X | Physician Assistants & Advanced Practice Nursing Providers; Nurse Practitioner; Pediatrics |
| 2083X0100X | Occupational Medicine |
| 208M00000X | Hospitalist |
| 2080A0000X | Adolescent Medicine |
| 2083P0500X | Preventative Medicine/Occupational Environmental Medicine |

© 2024 Rosen AK et al. *JAMA Health Forum*.

**eTable 4. Place of Service Claims Used to Identify Primary Care Utilization in Community Care Outpatient Claims**

| Place of Service Code | Definition |
|---|---|
| 2 | Telehealth |
| 3 | School |
| 4 | Homeless Shelter |
| 5 | Indian Health Service Free-standing Facility |
| 6 | Indian Health Service Provider-based Facility |
| 7 | Tribal 638 Free-standing Facility |
| 8 | Tribal 638 Provider-based Facility |
| 9 | Prison/Correctional Facility |
| 10 | Unassigned |
| 11 | Office |
| 12 | Home |
| 15 | Assisted Living Facility |
| 17 | Walk-in Retail Health Clinic |
| 18 | Place of Employment-Worksite |
| 19 | Off Campus-Outpatient Hospital |
| 20 | Urgent Care Facility |
| 22 | On Campus-Outpatient Hospital |
| 24 | Ambulatory Surgical Center |
| 26 | Military Treatment Facility |
| 27 | Unassigned |
| 28 | Unassigned |
| 29 | Unassigned |
| 30 | Unassigned |
| 35 | Unassigned |
| 36 | Unassigned |
| 37 | Unassigned |
| 38 | Unassigned |
| 39 | Unassigned |
| 40 | Unassigned |
| 43 | Unassigned |
| 44 | Unassigned |
| 45 | Unassigned |
| 46 | Unassigned |
| 47 | Unassigned |
| 48 | Unassigned |
| 49 | Independent Clinic |
| 50 | Federally Qualified Health Center |
| 52 | Psychiatric Facility-Partial Hospitalization |
| 53 | Community Mental Health Center |
| 55 | Residential Substance Abuse Treatment Facility |

© 2024 Rosen AK et al. *JAMA Health Forum*.

**eTable 4 (continued). Place of Service Claims Used to Identify Primary Care Utilization in Community Care Outpatient Claims**

| Place of Service Code | Definition |
|---|---|
| 56 | Psychiatric Residential Treatment Center |
| 57 | Non-residential Substance Abuse Treatment Facility |
| 59 | Unassigned |
| 62 | Comprehensive Outpatient Rehabilitation Facility |
| 63 | Unassigned |
| 64 | Unassigned |
| 71 | Public Health Clinic |
| 72 | Rural Health Clinic |
| 99 | Unassigned |

© 2024 Rosen AK et al. *JAMA Health Forum*.

**eTable 5. List and Definition of VA Stop Codes Excluded from Primary Care Utilization Measure (Do not Represent Primary Care Visits)**

| Stop Code | Definition |
|---|---|
| 108 | Laboratory |
| 119 | Community Nursing Home (CNH) Follow-Up |
| 121 | Community Residential Care (CRC) |
| 130 | Emergency Department |
| 190 | Adult Day Health Care (ADHC) (VA-based ADHC) |
| 191 | Community Adult Day Health Care (ADHC) Follow-Up |
| 213 | Physical Medicine and Rehabilitation Service (PM&RS) Vocational Assistance |
| 222 | Physical Medicine and Rehabilitation Service (PM&RS) Compensated Work Therapy/Supported Employment (PM&RS CWT/SE) Face-to-Face |
| 297 | Observation Emergency Room |
| 443 | Disability Benefits Questionnaire (DBQ) Referral Clinic |
| 444 | Compensation and Pension(C&P) Exam via Clinical Video Telehealth- Patient Site |
| 445 | Compensation and Pension (C&P) Exam via Clinical Video Telehealth- Provider Site |
| 446 | Integrated Disability and Evaluation System (IDES) Exam via Clinical Video Telehealth-Patient Site |
| 447 | Integrated Disability and Evaluation System (IDES) Exam via Clinical Video Telehealth-Provider Site |
| 448 | Integrated Disability Evaluation System Exam (IDES) |
| 450 | Compensation and Pension (C&P) Exam |
| 555 | Homeless Veteran Community Employment Services- Individual |
| 568 | Mental Health Compensated Work Therapy/Supported Employment (CWT/SE) Face-to-Face |
| 574 | Mental Health Compensated Work Therapy/Transitional Work Experience (CWT/TWE) Face-to-Face |
| 651 | State Nursing Home Days |
| 652 | State Residential Rehabilitation Treatment Program (RRTP) Home Days |
| 653 | State Hospital Care |
| 656 | Department of Defense (DOD) Non-VA Care |
| 658 | State Home Adult Day Health Care |
| 669 | Community Care Clinical Consult (Non-VA Care Clinical Consult) |
| 681 | VA-Paid Home-based Care (HCBC) Providers |
| 682 | VA-Referrals to Home-based Care (HCBC0 Providers |

© 2024 Rosen AK et al. *JAMA Health Forum.*

**eTable 6. List of Stopcodes Included for Measuring Primary Care Access**

| Stop Code | Stop Code Name |
|---|---|
| 322 | Comprehensive Women's and Gender Diverse Primary Care Clinic |
| 323 | Primary Care Medicine |
| 342 | Family Practice |
| 348 | Primary Care Shared Appointment |
| 350 | Geriatric Patient Aligned Care Team (GeriPACT) |
| 704 | Women's and Gender Diverse Gender-Specific Preventive Care |

© 2024 Rosen AK et al. *JAMA Health Forum.*

**eFigure 1. VA Community Care Regional Networks**

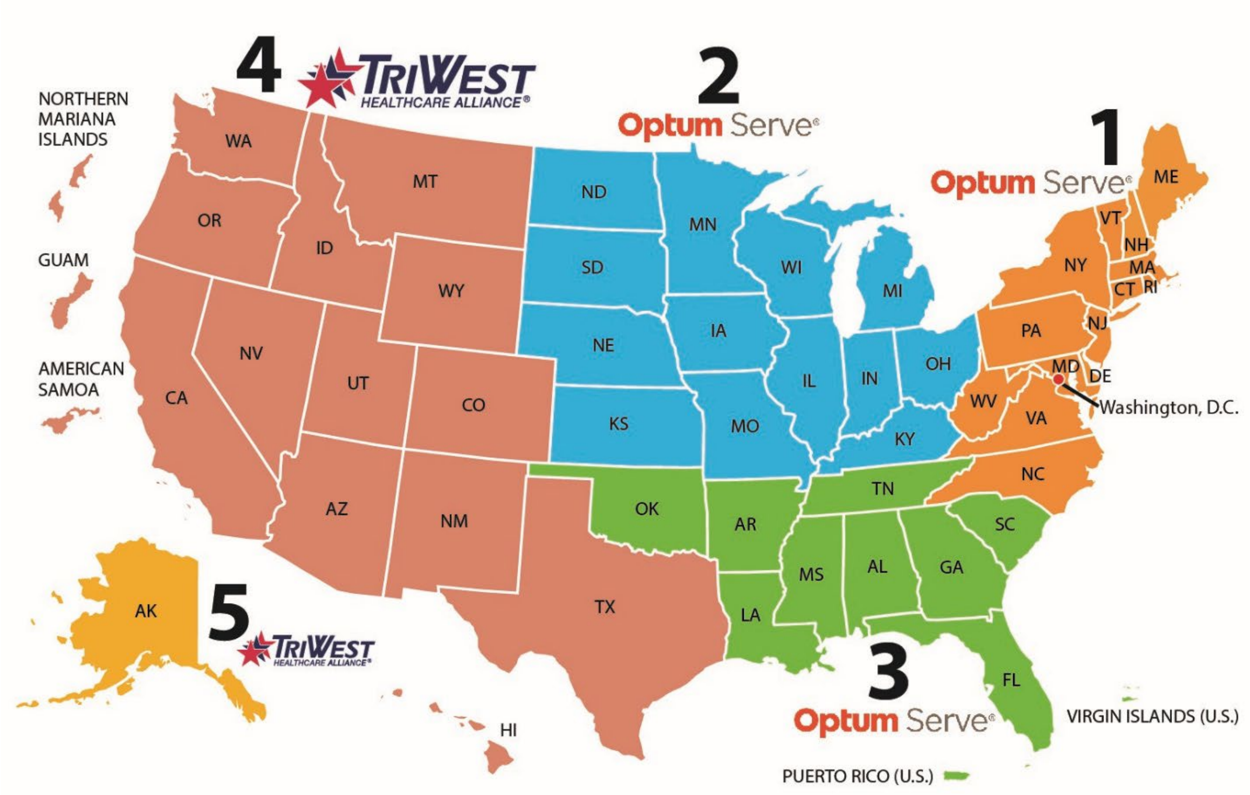


© 2024 Rosen AK et al. *JAMA Health Forum*.

## eTable 7. Utilization Cohort Demographics [a,b,c]

| | | Urban[d] | | | | | Rural[d] | | | | |
|---|---|---|---|---|---|---|---|---|---|---|---|
| | Overall | Black | Hispanic | White | Effect Size [p-value]: Blacks vs Whites | Effect Size [p-value]: Hispanics vs Whites | Black | Hispanic | White | Effect Size [p-value]: Blacks vs Whites | Effect Size [p-value]: Hispanics vs Whites |
| No. Distinct Veterans | 5046087 | 830574 | 331388 | 2160539 | NA [NA] | NA [NA] | 163943 | 59482 | 1500161 | NA [NA] | NA [NA] |
| Age, mean (SD) | 62.14 (16.54) | 57.93 (14.70) | 54.78 (17.75) | 63.19 (17.10) | 0.330 [<0.001] | 0.482 [<0.001] | 59.80 (13.99) | 56.79 (17.14) | 65.07 (15.58) | 0.356 [<0.001] | 0.505 [<0.001] |
| Gagne score, mean (SD) | 0.89 (2.01) | 0.86 (2.06) | 0.74 (1.77) | 0.93 (2.00) | 0.030 [<0.001] | 0.101 [<0.001] | 0.76 (2.00) | 0.67 (1.73) | 0.92 (2.05) | 0.08 [<0.001] | 0.133 [<0.001] |
| Priority Group, No. (%) | | | | | | | | | | | |
| Groups 1-3 | 3139634 (62.2) | 565387 (68.1) | 233234 (70.4) | 1285753 (59.5) | 0.179 [<0.001] | 0.229 [<0.001] | 114334 (69.7) | 42742 (71.9) | 898184 (59.9) | 0.208 [<0.001] | 0.255 [<0.001] |
| Groups 4-7 | 1261512 (25.0) | 194121 (23.4) | 72076 (21.8) | 572898 (26.5) | 0.073 [<0.001] | 0.112 [<0.001] | 33777 (20.6) | 11300 (19.0) | 377340 (25.2) | 0.108 [<0.001] | 0.149 [<0.001] |
| Group 8 | 644941 (12.8) | 71066 (8.6) | 26078 (7.9) | 301888 (13.9) | 0.172 [<0.001] | 0.197 [<0.001] | 15832 (9.7) | 5440 (9.2) | 224637 (14.9) | 0.162 [<0.001] | 0.18 [<0.001] |
| Sex, No. (%) | | | | | | | | | | | |
| Female | 4548411 (90.1) | 684858 (82.5) | 293343 (88.5) | 1977003 (91.5) | 0.271 [<0.001] | 0.100 [<0.001] | 22968 (14.0) | 6615 (11.1) | 100795 (6.7) | 0.241 [<0.001] | 0.155 [<0.001] |
| Male | 497675 (9.9) | 145716 (17.5) | 38045 (11.5) | 183536 (8.5) | 0.271 [<0.001] | 0.100 [<0.001] | 140974 (85.9) | 52867 (88.9) | 1399366 (93.3) | 0.241 [<0.001] | 0.155 [<0.001] |
| Unknown | 1 (0.0) | 0 (0.0) | 0 (0.0) | 0 (0.0) | NA [NA] | NA [NA] | 1 (0.0) | 0 (0.0) | 0 (0.0) | 0.003 [<0.001] | NA [NA] |
| Marital Status, No. (%) | | | | | | | | | | | |
| Married | 2808991 (55.7) | 358809 (43.2) | 177360 (53.5) | 1210435 (56.0) | 0.259 [<0.001] | 0.050 [<0.001] | 82640 (50.4) | 35600 (59.9) | 944147 (62.9) | 0.255 [<0.001] | 0.063 [<0.001] |
| Not Married | 2182300 (43.3) | 461934 (55.6) | 150963 (45.6) | 925051 (42.8) | 0.258 [<0.001] | 0.055 [<0.001] | 79433 (48.5) | 23272 (39.1) | 541647 (36.1) | 0.252 [<0.001] | 0.062 [<0.001] |
| Unknown | 54796 (1.1) | 9831 (1.2) | 3065 (0.9) | 25053 (1.2) | 0.002 [0.08] | 0.023 [<0.001] | 1870 (1.1) | 610 (1.0) | 14367 (0.9) | 0.018 [<0.001] | 0.007 [0.09] |
| Homeless/Homelessness-Risk, No. (%) | | | | | | | | | | | |
| | | | | | | | | | | | |

© 2024 Rosen AK et al. *JAMA Health Forum*.

## eTable 7 (continued). Utilization Cohort Demographics [a,b,c]

| | | Urban[d] | | | | | Rural[d] | | | | |
|---|---|---|---|---|---|---|---|---|---|---|---|
| | Overall | Black | Hispanic | White | Effect Size [p-value]: Blacks vs Whites | Effect Size [p-value]: Hispanics vs Whites | Black | Hispanic | White | Effect Size [p-value]: Blacks vs Whites | Effect Size [p-value]: Hispanics vs Whites |
| No | 4031013 (79.9) | 622757 (74.9) | 264759 (79.9) | 1709293 (79.1) | 0.098 [<0.001] | 0.019 [<0.001] | 135596 (82.7) | 49514 (83.2) | 1249094 (83.3) | 0.015 [<0.001] | 0.001 [0.888] |
| Yes | 115791 (2.3) | 38566 (4.6) | 9543 (2.9) | 42849 (1.9) | 0.149 [<0.001] | 0.058 [<0.001] | 4881 (2.9) | 1294 (2.2) | 18658 (1.2) | 0.121 [<0.001] | 0.072 [<0.001] |
| Unknown | 899283 (17.8) | 169251 (20.4) | 57086 (17.2) | 408397 (18.9) | 0.037 [<0.001] | 0.044 [<0.001] | 23466 (14.3) | 8674 (14.6) | 232409 (15.5) | 0.033 [<0.001] | 0.025 [<0.001] |
| Food Insecurity, No. (%) | | | | | | | | | | | |
| No | 3934193 (77.9) | 638123 (76.8) | 255883 (77.2) | 1651801 (76.5) | 0.009 [<0.001] | 0.018 [<0.001] | 133831 (81.6) | 47464 (79.8) | 1207091 (80.5) | 0.030 [<0.001] | 0.017 [<0.001] |
| Yes | 30419 (0.6) | 10351 (1.3) | 2746 (0.8) | 10198 (0.45 | 0.084 [<0.001] | 0.044 [<0.001] | 1365 (0.8) | 360 (0.6) | 5399 (0.4) | 0.061 [<0.001] | 0.035 [<0.001] |
| Unknown | 1081475 (21.4) | 182100 (21.9) | 72759 (21.9) | 498540 (23.1) | 0.028 [<0.001] | 0.027 [<0.001] | 28747 (17.5) | 11658 (19.6) | 287671 (19.2) | 0.042 [<0.001] | 0.011 [0.01] |
| Region, No. (%) | | | | | | | | | | | |
| Region 1 | 1142738 (22.7) | 215791 (25.9) | 38932 (11.8) | 507291 (23.5) | 0.058 [<0.001] | 0.312 [<0.001] | 40963 (24.9) | 5536 (9.3) | 334225 (22.3) | 0.064 [<0.001] | 0.361 [<0.001] |
| Region 2 | 1135219 (22.5) | 122930 (14.8) | 18611 (5.6) | 465051 (21.5) | 0.175 [<0.001] | 0.478 [<0.001] | 11357 (6.9) | 6444 (10.8) | 510826 (34.1) | 0.713 [<0.001] | 0.579 [<0.001] |
| Region 3 | 1412690 (28.0) | 298342 (35.9) | 98495 (29.772) | 561957 (26.0) | 0.216 [<0.001] | 0.083 [<0.001] | 92263 (56.3) | 10896 (18.3) | 350737 (23.4) | 0.713 [<0.001] | 0.125 [<0.001] |
| Region 4 | 1339387 (26.5) | 192324 (23.2) | 174900 (52.8) | 620300 (28.7) | 0.127 [<0.001] | 0.505 [<0.001] | 19097 (11.7) | 36343 (61.1) | 296423 (19.8) | 0.224 [<0.001] | 0.929 [<0.001] |
| Region 5 | 16053 (0.3) | 1187 (0.1) | 450 (0.1) | 5940 (0.3) | 0.029 [<0.001] | 0.031 [<0.001] | 263 (0.2) | 263 (0.4) | 7950 (0.5) | 0.063 [<0.001] | 0.013 [0.004] |

[a] Five VA facilities were excluded that had transiioned to Cerner, the new VA electronic health record system, due to lack of validated study measures at these sites.

[b] Veterans with unknown rurality status (9.3%) and islanders (0.04%) were excluded

[c] 328,501 patients (5%) were excluded from the utilization cohort because they were classifed as “unknown” (i.e., those cases that could not be classified due to missing race/ethnicity data, or reported as declined or unknown)

[d] Rurality was derived from the VA Planning Systems Support Group (PSSG), which utilizes the definition of rurality from the Office of Management and Budget (Urban, Rural, and Highly Rural) based on Rural-Urban Commuting Area codes.

© 2024 Rosen AK et al. *JAMA Health Forum.*

## eTable 8. Access Cohort Demographics [a,b,c]

| | | Urban[d] | | | | | Rural[d] | | | | |
|---|---|---|---|---|---|---|---|---|---|---|---|
| | Overall | Black | Hispanic | White | Effect Size [p-value]: Blacks vs Whites | Effect Size [p-value]: Hispanics vs Whites | Black | Hispanic | White | Effect Size [p-value]: Blacks vs Whites | Effect Size [p-value]: Hispanics vs Whites |
| No. Distinct Veterans | 386764 | 67977 | 28845 | 153826 | NA [NA] | NA [NA] | 11828 | 4946 | 119342 | NA [NA] | NA [NA] |
| Age, mean (SD) | 61.14 (16.69) | 57.18 (15.25) | 58.72 (18.13) | 61.33 (17.24) | 0.255 [<0.001] | 0.148 [<0.001] | 58.38 (14.67) | 56.64 (16.88) | 64.19 (15.90) | 0.380 [<0.001] | 0.460 [<0.001] |
| Gagne score, mean (SD) | 0.94 (2.01) | 0.90 (2.02) | 0.88 (1.94) | 0.95 (2.00) | 0.025 [<0.001] | 0.036 [<0.001] | 0.83 (1.97) | 0.75 (1.77) | 0.99 (2.05) | 0.083 [<0.001] | 0.129 [<0.001] |
| Priority Group, No. (%) | | | | | | | | | | | |
| Groups 1-3 | 250300 (64.7) | 47103 (69.3) | 20109 (69.7) | 95758 (62.3) | 0.149 [<0.001] | 0.158 [<0.001] | 8557 (72.4) | 3694 (74.7) | 75079 (62.9) | 0.203 [<0.001] | 0.256 [<0.001] |
| Groups 4-7 | 97674 (25.3) | 15896 (23.4) | 7059 (24.5) | 41634 (27.1) | 0.085 [<0.001] | 0.059 [<0.001] | 2312 (19.6) | 891 (18.0) | 29882 (25.0) | 0.132 [<0.001] | 0.172 [<0.001] |
| Group 8 | 38790 (10.0) | 4978 (7.3) | 1677 (5.8) | 16434 (10.7) | 0.118 [<0.001] | 0.178 [<0.001] | 959 (8.1) | 361 (7.3) | 14381 (12.1) | 0.131 [<0.001] | 0.161 [<0.001] |
| Sex, No. (%) | | | | | | | | | | | |
| Female | 318758 (82.4) | 47512 (69.9) | 23507 (81.5) | 129159 (83.9) | 0.339 [<0.001] | 0.065 [<0.001] | 8935 (75.5) | 3999 (80.9) | 105646 (88.5) | 0.343 [<0.001] | 0.214 [<0.001] |
| Male | 68006 (17.6) | 20465 (30.1) | 5338 (18.5) | 24667 (16.0) | 0.339 [<0.001] | 0.065 [<0.001] | 2893 (24.5) | 947 (19.2) | 13696 (11.5) | 0.343 [<0.001] | 0.214 [<0.001] |
| Marital Status, No. (%) | | | | | | | | | | | |
| Married | 195276 (50.5) | 25549 (37.6) | 14644 (50.8) | 77040 (50.1) | 0.254 [<0.001] | 0.014 [0.033] | 5427 (45.9) | 2866 (57.9) | 69750 (58.5) | 0.253 [<0.001] | 0.010 [0.485] |
| Not Married | 180414 (46.7) | 40287 (59.3) | 13099 (45.4) | 72551 (47.2) | 0.244 [<0.001] | 0.035 [<0.001] | 6049 (51.1) | 1933 (39.1) | 46495 (38.9) | 0.247 [<0.001] | 0.003 [0.86] |
| Unknown | 11074 (2.9) | 2141 (3.2) | 1102 (3.8) | 4235 (2.8) | 0.023 [<0.001] | 0.06 [<0.001] | 352 (2.9) | 147 (2.9) | 3097 (2.6) | 0.023 [0.01] | 0.023 [0.10] |
| Homeless/Homelessness -Risk, No. (%) | | | | | | | | | | | |
| No | 268516 (69.4) | 46633 (68.6) | 23059 (79.9) | 105472 (68.6) | 0.001 [0.87] | 0.262 [<0.001] | 8075 (68.3) | 3568 (72.1) | 81709 (68.5) | 0.004 [0.66] | 0.080 [<0.001] |
| Yes | 21656 (5.6) | 7958 (11.7) | 1433 (4.9) | 8081 (5.3) | 0.233 [<0.001] | 0.013 [0.05] | 776 (6.6) | 178 (3.6) | 3230 (2.7) | 0.184 [<0.001] | 0.051 [<0.001] |

© 2024 Rosen AK et al. *JAMA Health Forum*.

**eTable 8 (continued). Access Cohort Demographics [a,b,c]**

| | | | | | | | | | | | |
|---|---|---|---|---|---|---|---|---|---|---|---|
| Unknown | 96592 (24.9) | 13386 (19.7) | 4353 (15.1) | 40273 (26.2) | 0.155 [<0.001] | 0.277 [<0.001] | 2977 (25.2) | 1200 (24.3) | 34403 (28.8) | 0.082 [<0.001] | 0.104 [<0.001] |
| Food Insecurity, No. (%) | | | | | | | | | | | |
| No | 245430 (63.5) | 44356 (65.3) | 21464 (74.4) | 94918 (61.7) | 0.074 [<0.001] | 0.275 [<0.001] | 7492 (63.3) | 3283 (66.4) | 73917 (61.9) | 0.029 [0.003] | 0.093 [<0.001] |
| Yes | 2017 (0.5) | 718 (1.1) | 233 (0.8) | 633 (0.4) | 0.076 [<0.001] | 0.051 [<0.001] | 75 (0.6) | 25 (0.5) | 333 (0.3) | 0.053 [<0.001] | 0.036 [<0.001] |
| Unknown | 139317 (36.0) | 22903 (33.7) | 7148 (24.8) | 58275 (37.9) | 0.088 [<0.001] | 0.285 [<0.001] | 4261 (36.0) | 1638 (33.1) | 45092 (37.8) | 0.036 [<0.001] | 0.098 [<0.001] |
| Community Care Network (CCN) Region, No. (%) | | | | | | | | | | | |
| CCN Region 1 | 95457 (24.7) | 19599 (28.8) | 3442 (11.9) | 39451 (25.7) | 0.072 [<0.001] | 0.357 [<0.001] | 4100 (34.7) | 501 (10.1) | 28364 (23.8) | 0.241 [<0.001] | 0.370 [<0.001] |
| CCN Region 2 | 100333 (25.9) | 13779 (20.3) | 1558 (5.4) | 41239 (26.8) | 0.155 [<0.001] | 0.609 [<0.001] | 1077 (9.1) | 574 (11.6) | 42106 (35.3) | 0.664 [<0.001] | 0.582 [<0.001] |
| CCN Region 3 | 86719 (22.4) | 21495 (31.6) | 13198 (45.8) | 28513 (18.5) | 0.305 [<0.001] | 0.609 [<0.001] | 5298 (44.8) | 726 (14.7) | 17489 (14.7) | 0.698 [<0.001] | 0.001 [0.96] |
| CCN Region 4 | 101470 (26.2) | 12875 (18.9) | 10567 (36.6) | 43657 (28.4) | 0.224 [<0.001] | 0.177 [<0.001] | 1302 (11.0) | 3100 (62.7) | 29969 (25.1) | 0.373 [<0.001] | 0.818 [<0.001] |
| CCN Region 5 | 2785 (0.7) | 229 (0.3) | 80 (0.3) | 966 (0.6) | 0.042 [<0.001] | 0.052 [<0.001] | 51 (0.4) | 45 (0.9) | 1414 (1.2) | 0.084 [<0.001] | 0.027 [0.08] |

[a] Five VA facilities were excluded that had transiioned to Cerner, the new VA electronic health record system, due to lack of validated study measures at these sites.
[b] Veterans with unknown rurality status (3.9%) and islanders (0.08%) were excluded
[c] 31,880 patients (7%) were excluded from the access cohort because they were classifed as “unknown” (i.e., those cases that could not be classified due to missing race/ethnicity data, or reported as declined or unknown)
[d] Rurality was derived from the VA Planning Systems Support Group (PSSG), which utilizes the definition of rurality from the Office of Management and Budget (Urban, Rural, and Highly Rural) based on Rural-Urban Commuting Area codes.

© 2024 Rosen AK et al. *JAMA Health Forum*.
