# Supplementary material for: Racial and Ethnic and Rural Variations in Access to Primary Care for Veterans Following the MISSION Act
Source: JAMA Health Forum. 2024 Jun 21;5(6):e241568. doi: 10.1001/jamahealthforum.2024.1568 (PMC11193128; doi:10.1001/jamahealthforum.2024.1568)
Supplement: Supplement 2. — Data sharing statement [file jamahealthforum-e241568-s002.pdf]

Data Sharing Statement

Rosen. Racial and Ethnic and Rural Variations in Access to Primary Care for Veterans Following the MISSION Act. *JAMA Health Forum*. Published June 21, 2024. doi:10.1001/jamahealthforum.2024.1568

Data
Data available: No
